# Supplementary material for: Impact of Chronic Obstructive Pulmonary Disease on Incidence, Microbiology and Outcome of Ventilator-Associated Lower Respiratory Tract Infections
Source: Microorganisms. 2020 Jan 23;8(2):165. doi: 10.3390/microorganisms8020165 (PMC7074722; doi:10.3390/microorganisms8020165)
Supplement: Supplementary file 1 [file microorganisms-08-00165-s001.pdf]

SUPPLEMENTARY MATERIAL

Table S1. Risk factors for ICU mortality in patients with ventilator-associated lower respiratory tract infection.

	**Univariate analyses**		**Multivariate analysis***	
	**OR (95% CI)**	**p-value**	**OR (95% CI)**	**p-value**
Age, per year	1.03 (1.02 to 1.04)	**<0.001**	1.03 (1.02 to 1.04)	**<0.001**
Male gender	0.90 (0.65 to 1.26)	0.54		
SAPS II, per point	1.02 (1.01 to 1.03)	**<0.001**	-	-
SOFA score at VA-LRTI diagnosis, per point	1.20 (1.15 to 1.26)	**<0.001**	1.21 (1.14 to 1.27)	**<0.001**
Diabetes mellitus	1.49 (1.02 to 2.20)	**0.041**	-	-
Chronic respiratory failure	2.89 (1.70 to 4.92)	**<0.001**	2.22 (1.21 to 4.07)	**0.010**
Chronic heart failure	1.49 (0.81 to 2.73)	0.20		
Chronic kidney disease	3.34 (2.00 to 5.57)	**<0.001**	1.89 (1.04 to 3.43)	**0.036**
Cirrhosis	2.08 (1.01 to 4.29)	**0.047**	-	-
Immunosuppression	2.68 (1.79 to 4.02)	**<0.001**	2.13 (1.34 to 3.36)	**0.001**
Alcohol abuse	1.52 (0.95 to 2.42)	0.078	-	-
Intravenous drug abuse	0.70 (0.19 to 2.67)	0.60		
**COPD**	1.38 (0.93 to 2.05)	0.11		
Multidrug-resistant isolates	1.72 (1.23 to 2.40)	**0.002**	1.53 (1.05 to 2.23)	**0.026**
Antibiotic treatment	1.07 (0.49 to 2.31)	0.87		
Appropriate antibiotic treatment	0.57 (0.37 to 0.87)	**0.009**	0.48 (0.29 to 0.79)	**0.004**
VAP	1.60 (1.16 to 2.20)	**0.004**	1.57 (1.07 to 2.31)	**0.021**
VAT to VAP progression**	1.84 (0.92 to 3.67)	0.082		

COPD, Chronic Obstructive Pulmonary Disease; ICU, Intensive Care Unit; OR, odds-ratio; CI, confidence interval; SAPS, Simplified Acute Physiology Score; SOFA, Sequential Organ Failure Assessment; VA-LRTI, ventilator-associated lower respiratory tract infection; VAP, ventilator-associated pneumonia; VAT, ventilator-associated tracheobronchitis.

* Factor with a p-value < 0.10 in univariate analyses (excepted VAT to VAP progression) were included into a stepwise backward logistic regression model

** Only available for VAT population (n=320)
